# Machine Learning Enables High-Throughput Phenotyping for Analyses of the Genetic Architecture of Bulliform Cell Patterning in Maize

**DOI:** 10.1534/g3.119.400757

**Published:** 2019-10-23

**Authors:** Pengfei Qiao, Meng Lin, Miguel Vasquez, Susanne Matschi, James Chamness, Matheus Baseggio, Laurie G. Smith, Mert R. Sabuncu, Michael A. Gore, Michael J. Scanlon

**Affiliations:** * Plant Biology Section, School of Integrative Plant Science,; † Plant Breeding and Genetics Section, School of Integrative Plant Science,; § School of Electrical and Computer Engineering, Cornell University, Ithaca, NY 14853, and; ‡ Section of Cell and Developmental Biology, University of California San Diego, La Jolla, 92093, and

**Keywords:** Machine learning, development, bulliform cell, GWAS

## Abstract

Bulliform cells comprise specialized cell types that develop on the adaxial (upper) surface of grass leaves, and are patterned to form linear rows along the proximodistal axis of the adult leaf blade. Bulliform cell patterning affects leaf angle and is presumed to function during leaf rolling, thereby reducing water loss during temperature extremes and drought. In this study, epidermal leaf impressions were collected from a genetically and anatomically diverse population of maize inbred lines. Subsequently, convolutional neural networks were employed to measure microscopic, bulliform cell-patterning phenotypes in high-throughput. A genome-wide association study, combined with RNAseq analyses of the bulliform cell ontogenic zone, identified candidate regulatory genes affecting bulliform cell column number and cell width. This study is the first to combine machine learning approaches, transcriptomics, and genomics to study bulliform cell patterning, and the first to utilize natural variation to investigate the genetic architecture of this microscopic trait. In addition, this study provides insight toward the improvement of macroscopic traits such as drought resistance and plant architecture in an agronomically important crop plant.

Drought stress remains a serious challenge to agronomic production (Ort and Long 2014); land plants have evolved multiple mechanisms for water conservation since their invasion of the terrestrial environment more than 450 million years ago (Kenrick and Crane 1997; Raven and Edwards 2004). Grasses are staple crops for human subsistence and have evolved specific epidermal cell types (*i.e.*, bulliform cells) to reduce water loss during heat and drought (Hsiao *et al.* 1984; Price *et al.* 1997; Kadioglu and Terzi 2007; Hu *et al.* 2010). Bulliform cells are enlarged parenchymatous structures arranged in tandem clusters that form linear columns along the proximodistal leaf axis (Becraft *et al.* 2002; Bennetzen and Hake 2008). During heat and/or water stress, bulliform cells are proposed to shrink dramatically in size along the adaxial (top) leaf surface. This asymmetric decrease in leaf surface area is a proposed mechanism for leaf rolling, consequently reducing water loss from the leaf epidermis (Hsiao *et al.* 1984; Price *et al.* 1997; Dai *et al.* 2007; Kadioglu and Terzi 2007; Hu *et al.* 2010). Some bulliform cell number and density mutants also have leaf angle phenotypes, thus impacting plant architecture. Rice bulliform cell patterning mutants such as *RICE OUTERMOST CELL-SPECIFIC GENE5 (Roc5)* over-produce bulliform cells, have more upright leaves, which is a desirable agronomic trait enabling dense planting (Zou *et al.* 2011).

Despite the inherent interest in bulliform cell patterning to both plant developmental biologists and breeders, previous studies have focused on either the cell-specific transcriptomes or reverse genetics analyses of mature-staged bulliform cells. For example, a study in rice showed that bulliform cells express around 16,000 genes, far more than the median of 8,831 genes identified in RNAseq analyses of over 40 distinct cell types (Jiao *et al.* 2009). Coincidentally, reverse genetic studies reveal that mutations in genes implicated in a diverse array of biological processes can condition bulliform cell phenotypes. For example, the brassinosteroid phytohormones, gibberellin and auxin, both function during bulliform cell patterning in rice (Dai *et al.* 2007; Fujino *et al.* 2008; Chen *et al.* 2015), whereas some leaf-rolling mutants have supernumerary bulliform cells and others develop ectopic bulliform cells on the abaxial (bottom) side of the leaf (Itoh *et al.* 2008; Hibara *et al.*, 2009: Zhang *et al.* 2009; Li *et al.* 2010). Aside from defects in adaxial/abaxial patterning, some leaf rolling mutants are also impaired in water transport (Fang *et al.* 2012), or in the production of a vacuolar ATPase (Xiang *et al.* 2012). Despite these genetic analyses of bulliform development, no studies have been performed on the natural variation of bulliform cell patterning in a staple crop plant such as maize.

Elucidating the genetic architecture controlling natural variation of maize bulliform cell patterning is fraught with challenges. Although bulliform cells influence a wide range of macroscopic traits such as leaf rolling and leaf angle, bulliform cell patterning is a microscopic phenotype. Historically, epidermal cells are typically analyzed by scanning electron microscopy (SEM) (Becraft *et al.* 2002), or light-imaging of epidermal glue-impressions (Bennetzen and Hake 2008). Although SEM is not amenable to high-throughput phenotyping of large plant populations, epidermal glue-impressions are relatively easy to generate in high volume and can be stored for extended periods, thereby preserving cellular structures in great detail (Bennetzen and Hake 2008).

Another bottleneck to high-throughput phenotyping of microscopic epidermal traits is the quantification of cell profiles *after* image acquisition. Machine learning strategies such as convolutional neural networks (CNNs) are widely used for image processing; advances in modern technology have enabled the optimization of complex machine learning models comprising millions of parameters (LeCun and Bengio 1995; LeCun *et al.*, 1998: Krizhevsky *et al.* 2012; Simonyan and Zisserman 2014; Zeiler and Fergus 2014; Szegedy *et al.* 2015; He *et al.* 2016). Semantic segmentation of microscopic images via CNNs can significantly decrease the labor and time required to manually score such phenotypes in large-scale genetic studies. Special CNN algorithms such as U-net enable the efficient use of context information of image pixels, thereby reducing the otherwise daunting workload of manually tracing cell anatomical patterns into a matter of seconds (Ronneberger *et al.* 2015).

In this study, leaf epidermal glue-impressions were collected from a genetically diverse panel of nearly 500 maize inbred lines, and U-nets were utilized to quantify bulliform cell patterning phenotypes from over 60,000 leaf images within this population. A genome-wide association study (GWAS) (Yu *et al.* 2006; Lipka *et al.* 2012) was then performed to identify loci associated with bulliform cell column number and width. In addition, the ontogeny of bulliform cell development in the expanding maize leaf was analyzed, which informed the stage-specific isolation of mRNA from the region of bulliform cell initiation and differentiation in the developing maize leaf. Considering both these GWAS and transcriptomic data, we propose candidate genes responsible for bulliform cell patterning in maize.

## Materials and Methods

### Bulliform cell ontogeny and RNA sequencing

Seeds of maize inbred line B73 (accession number: PI 550473) were obtained from the Maize Genetics Cooperation Stock Center. Three replicates of B73 plants were grown in Percival A100 growth chambers with 10-hour day length at temperatures 25° day, 20° night, and relative humidity of 60%. Plants were grown for 33 days, when the partially elongated leaf eight was 50-55 cm long. Leaf eight was dissected out of the whorl and EXAFLEX Vinyl Polysiloxane Impression Material (Injectable) was applied onto the basal 5 cm of the blade to make epidermal glue-impressions.

Total RNA was isolated from the 0 – 2 mm region distal to the ligule of the expanding leaf 8 using the TRIzol Reagent in three replicates. The NEBNext Ultra RNA Library Prep Kit for Illumina was used to construct sequencing libraries. The Illumina HiSEQ 2500 instrument was used for 150 bp paired-end sequencing. After sequencing, reads were aligned to B73 version 4 genome with HiSAT2 (Kim *et al.* 2015) and counted with HTSeq (Anders *et al.* 2015).

### Differential gene expression analysis

Differential gene expression analysis was performed in R with the edgeR 3.3.2 package (Robinson *et al.* 2010; McCarthy *et al.* 2012) comparing the transcriptomes of the 2 mm and 15 - 35 mm regions distal to the ligule. Gene expression levels were normalized against library sizes. The default generalized linear model was used to call differential expressions. Genes with false discovery rate (FDR) less than 0.10 were declared as being significantly differentially expressed.

### Experimental design

A set of 468 maize inbred lines sampled from the Wisconsin Diversity (WiDiv) panel (Hirsch *et al.* 2014) (Table S1) were evaluated for bulliform cell patterning traits in adult leaves. The inbred lines were planted at the Maricopa Agricultural Center, Maricopa, AZ, and the University of California San Diego, San Diego, CA in 2017. The layout of the experiment in each location was arranged as an 18 × 26 incomplete block design (Table S2 – S3). Each incomplete block of 18 experimental lines was augmented by the random positioning of two check inbred lines (N28HT and Mo17). The entire experiment of 468 unique inbred lines plus checks was grown as a single replicate in each location. Edge effects were reduced by planting border maize plants around the perimeter of each replicate. Experimental units were one-row plots of 3.05 m (Maricopa) and 4.88 m (San Diego) in length with 1.02 m inter-row space. At the end of each plot there was a 0.91 m alley. Twelve kernels were planted in each plot, which were later thinned as needed.

### Leaf epidermal phenotypic data collection

Plants were grown in two environments under standard agronomic practices during the summer of 2017: San Diego, CA and Maricopa, AZ. To help control for differential rates of plant development, we scored flowering time (days to anthesis) as the total number of days from planting to the start of pollen shed for 50% of plants/plot. Leaf samples were taken from five plants per inbred line (plot), when at least half of the plants in that plot were at anthesis. Each leaf sample was taken midway between the ligule and the tip of the blade of the primary ear node leaf, or from one leaf younger. Midrib and margins were removed from the leaf sample to ensure that all samples were derived from the mid-blade. After harvesting, leaf samples were stored in Ziploc bags filled with water overnight at 4°, to ensure full hydration of epidermal cells and to capture an accurate representation of bulliform cell patterning under hydrated conditions. Following hydration, leaf samples were pressed onto slides with Loctite Super Glue Liquid Professional to generate leaf epidermal glue-impressions. Leaf glue-impressions were air-dried for at least 10 min, and removed from the leaf surfaces. Leaf epidermal glue-impressions were stored on slides at room temperature for future imaging. For each glue impression, three RGB images sampling different areas of the impression were taken with a Zeiss Z1/ApoTome stereo-microscope in bright field using a 1X objective lens.

### Neural networks in the quantification of phenotypic data

Convolutional neural networks (CNNs) were employed to quantify bulliform cell patterning traits in leaf epidermal glue-impression images. Each image was first resized to a 968 × 1292 grayscale image using Python module skimage 0.14.2 and cropped to the shape of 960 × 960 with Python module numpy 1.16.3. Each image was further split into four 480 × 480 images for faster computation. A training and validation set of 120 randomly sampled images and a test set of 20 randomly sampled images were created by manually annotating the pixels that are bulliform cells with Python module OpenCV 3.3.0 and skimage 0.14.2. Five U-nets were trained on 120 training images in Python with modules Keras 2.2.4 and TensorFlow 1.10.0.

In the U-nets, a contracting phase and expanding phase were included as described (Ronneberger *et al.* 2015). The contracting phase comprised repeated units of two convolution layers and one maxpooling layer, and the expanding phase included repeated units of two convolution layers and one up-convolution layer, after which the input dimensions were eventually restored.

The output of five U-nets was aggregated as the finalized output segmentation map by taking the average of the model output for each pixel. After segmentation, every four 480 × 480 images were put back to their original 960 × 960 images to quantify the bulliform patterning phenotypes.

Ten percent of the 120 training images were used as the validation set to determine the optimal learning rate of $5\;\times 10^{-5}$ (different learning rates and their associated losses are shown in Figure S1). Binary cross entropy was used as the loss function for the training, validation, and test set. Trained models are included in File S1. The output of five U-nets was aggregated as the finalized output segmentation map. After segmentation, every four 480 × 480 images were put back to their original 960 × 960 images to quantify the bulliform patterning phenotypes.

Each segmentation map is a two-dimensional array with binary elements. The two bulliform cell patterning phenotypes: bulliform cell column number and width, were quantified as below. In cases where there were more than three continuous pixels classified as bulliform cells, one column of bulliform cells was counted. The ratio between the total number of pixels annotated as bulliform cells and the number of bulliform cell columns is the average bulliform cell width of the image. To acquire model accuracies in regard to the bulliform cell patterning traits, a separate set of 30 images were manually annotated and model accuracies were derived by comparing the CNN-generated segmentation map and the manual annotation.

### Statistical data analysis

To screen the phenotypic data (bulliform column width, bulliform column number, or flowering time) for significant outliers, univariate mixed linear models were fitted as follows: (1) each single environment; and (2) both environments. The model terms included grand mean and check as fixed effects and environment, genotype, genotype-by-environment (G×E) interaction (only for models ii), incomplete block within environment, and column within environment as random effects. The Studentized deleted residuals (Kutner *et al.* 2005) generated from these mixed linear models were assessed and significant (α = 0.05) outliers removed. For each outlier screened phenotype, an iterative mixed linear model fitting procedure was conducted for each of the two full models in ASReml-R version 3.0 (Butler *et al.* 2009). All random terms that were not significant at α = 0.05 in a likelihood ratio test were removed from the model, allowing a final best-fit model to be obtained for each phenotype. These final models were used to generate a best linear unbiased predictor (BLUP) for each line (Table S4 – S10).

Variance component estimates from the fitted mixed linear models (Tables S11 – S16) were used for the estimation of heritability (Holland *et al.* 2003; Hung *et al.* 2012) for each phenotype within (plot basis) and across (line-mean basis) environments. Standard errors of the heritability estimates were calculated with the delta method (Holland *et al.* 2003; Lynch and Walsh 1998).

### DNA extraction, genotyping and SNP identification

For each of the 468 inbred lines in the WiDiv panel, total genomic DNA was extracted from a bulk of young leaves from a single plant. The leaf tissue samples were lyophilized and ground using a GenoGrinder (Spex SamplePrep, Metuchen, NJ, USA), followed by the isolation of genomic DNA using the DNeasy 96 Plant Kit (Qiagen Inc., Valencia, CA, USA). DNA samples were sent for genotyping-by-sequencing (GBS) (Elshire *et al.* 2011) at the Cornell Biotechnology Resource Center (Cornell University, Ithaca, NY, USA) with restriction enzyme *Ape*KI. GBS libraries were constructed and multiplexed 192-fold for sequencing on an Illumina NextSeq 500 instrument.

Genotypes at 955,690 high-confidence single-nucleotide polymorphism (SNP) loci were called with B73 RefGen_v2 coordinates as described (Baseggio *et al.* 2019). The raw SNP genotype calls were filtered to discard singleton and doubleton SNPs (a minor allele observed in a single line), and only biallelic SNPs with call rates greater than 40% and minimum inbreeding coefficient of 0.8 were retained. Missing SNP genotypes were partially imputed using FILLIN (Swarts *et al.* 2014) with a set of maize haplotype donor files with a 4 kb window size (AllZeaGBSv2.7impV5_AnonDonors4k.tar.gz, available at panzea.org). Physical coordinates of the SNP loci were uplifted to B73 RefGen_AGPv4. To uplift physical coordinates of the SNP loci to B73 RefGen_AGPv4, a 101 bp flanking sequence for each SNP (+/− 50 bp from a SNP) was aligned to B73 RefGen_AGPv4 using Vmatch (Kurtz 2003) to obtain the uplifted SNP coordinates. SNPs with flanking sequences that could not be uniquely and perfectly aligned to the reference genome were removed from the dataset. The final complete set contained 258,690 SNP markers.

### Genome-wide association study

Identified SNPs with minimum minor allele counts of 40 (4.28% minor allele frequency), minimum call rates of 60%, maximum heterozygosity of 10%, and a minimal inbreeding coefficient of 0.8 were retained, resulting in 258,308 high-quality GBS SNP markers (Table S18). After the removal of low-quality images and outliers, 461 inbred lines remained for use in GWAS in each environment and across both environments. For each bulliform cell patterning trait, a univariate mixed linear model was used with R package GAPIT 3.0 enabling Population Parameters Previously Determined (P3D) to conduct the GWAS (Zhang *et al.* 2010; Lipka *et al.* 2012). A subset of 41,259 SNPs remaining after linkage disequilibrium (LD) pruning (*r^2^* ≤ 0.2) of the complete marker data set in PLINK version 1.09_beta5 (Purcell *et al.* 2007) was used to calculate the genomic relationship (kinship) matrix. The kinship matrix was calculated with the VanRaden method included in the GAPIT package with no compression used when conducting GWAS. Flowering time BLUP values (Table S17) included to reduce the confounding influence of flowering time when detecting marker-trait associations and estimating allelic effects, together with up to ten PCs calculated from the SNP genotype matrix (Table S18) to control population structure, were tested as covariates using the Bayesian information criterion in the GAPIT package; only flowering time was selected for the GWAS models of both buliform traits, in all tested single and multiple environments. In GWAS, we found that our implemented univariate mixed linear model to be superior to a multivariate mixed linear model that modeled genotype-by-environment interactions (data not shown), thus only results from the univariate GWAS are reported. To control for the multiple testing problem, the false-discovery rate (FDR) was calculated as described in the Benjamini-Hochberg method (Benjamini and Hochberg 1995). Significant associations between the trait BLUPs and SNPs were tested and reported at the 5% FDR level.

### Linkage disequilibrium analysis

Linkage disequilibrium (LD) was estimated with squared allele frequency correlations (*r^2^*) as described (Lewontin 1988). For each top (*i.e.*, most significant) SNP at a locus, *r^2^* between all the other SNPs on the same chromosome and the top SNP were calculated, and genes that reside in a window spanned by SNPs in stronger than 0.5 LD with the top SNP were investigated as putative candidate genes.

### Data availability

The raw GBS sequencing data were deposited at NCBI SRA with accession number SRP160407 and in BioProject under accession PRJNA489924. The raw RNAseq data were deposited at NCBI SRT with SRA accession numbers PRJNA545465 and PRJNA400334. Leaf epidermal glue-impression images can be found at https://de.cyverse.org/dl/d/8CA8D72B-24AF-4887-8899-14460021887A/resized.zip. The scripts including LD calculation, image processing, U-net architecture, and running the GWAS are deposited in https://github.com/pengfei-qiao/Bulliform-cell-deep-learning.git. Trained U-net models are deposited as File S1 under https://de.cyverse.org/dl/d/B352A862-5B08-4373-87EB-9B48356028C6/FlieS1.zip. We request that this manuscript be cited when using these data. Supplemental material available at figshare: https://doi.org/10.25387/g3.9939623.

## Results and Discussion

### Bulliform cell ontogeny

**Figure 1:**
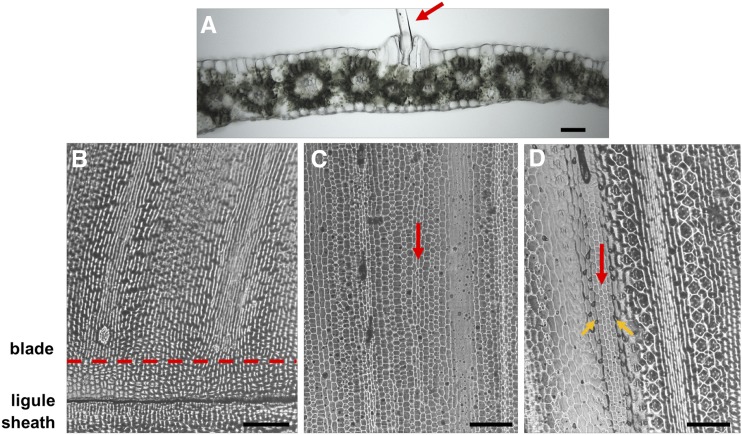
Ontogeny of maize bulliform cell development. (A) A mature bulliform cell cluster containing four morphologically distinct cells and a macrohair on the adaxial surface of an adult leaf. (B) No differences in cellular morphology are detectable in a 2 mm region of blade immediately distal to the ligule (bounded by the dashed red line) of the emerging adult leaf 8. (C) 15 mm region distal to the ligule, showing differences in cell morphology in files of cell columns, but no distinguishing bulliform cell characteristics. (D) 30 mm region distal to the ligule. The red arrow in (D) marks the same bulliform cell column denoted by the red arrow in (C), which indicates the cell column in (C) is an early stage of bulliform cell ontogeny. Orange arrows denote prickle hairs flanking bulliform cells. Scale bar 200 μm in (A), 2 mm in (B-D).

The strap-like maize leaf is composed of the proximal sheath and the distal blade, which are separated by the ligule/auricle blade-sheath boundary (Figure 1). The sheath surrounds the stem and inserts at the node, whereas the blade extends away from the stem and is the major photosynthetic portion of the leaf. Bulliform cells are found only on the adaxial leaf blade, forming clusters that are 4-5 cells wide and arranged in linear columns that extend the length of the blade, in parallel to the lateral veins (Figure 1). Macrohairs are specialized hairs that develop in the center of the bulliform cell rows (Figure 1A).

The ontogeny of bulliform cells was investigated in order to generate an RNA sequencing (RNAseq) library from the site of bulliform cell initiation, to be used as a crosscheck of our GWAS candidate genes for bulliform cell patterning. At 33 days after planting, the maize B73 adult leaf number 8 is still elongating from a meristematic region near the base of the leaf blade, just distal to the ligule as shown in Figure 1B. Epidermal impressions near the proximal end of the leaf blade, approximately 2 mm distal to the ligule of maize leaf eight, show no morphological evidence of bulliform cell patterning (Figure 1B). Approximately 15 mm from the ligule, morphological differences in epidermal cells are observed (Figure 1C), although bulliform cells are not yet distinguishable. Thirty mm beyond the ligule, however, cell types such as prickle hairs and bulliform cells are identified by their distinctive morphologies (Figure 1D). Thus, by proximally tracking bulliform cell rows that are visible at 30 mm from the ligule down to 15 mm from the ligule and lower, it is possible to identify immature bulliform cell rows before they develop their distinctive morphology. These analyses of epidermal cell development suggest that the bulliform cell ontogenic zone of the expanding leaf 8, where developmental patterning of the bulliform cells begins, is located approximately 2 mm above the ligule (Figure 1B).

RNAseq was performed on leaf tissue harvested from the bulliform cell ontogenic zone (Figure 1B). A differential gene expression analysis comparing the transcriptomes of the bulliform cell ontogenic zone and that of a distal blade interval harvested from 15 -35mm above the ligule of leaf 8 was conducted. Using an FDR of < 0.10, 15,081 out of 18,264 total transcripts were differentially expressed in the bulliform cell ontogenic zone as compared to more the distal, differentiated leaf tissues (Table S19). These data suggest that bulliform cell patterning is regulated by a complex transcriptomic network. Importantly, this tissue-specific dataset provides a unique resource toward the selection of candidate genes contributing to bulliform cell patterning.

### Phenotype variability and phenotyping accuracy

**Figure 2:**
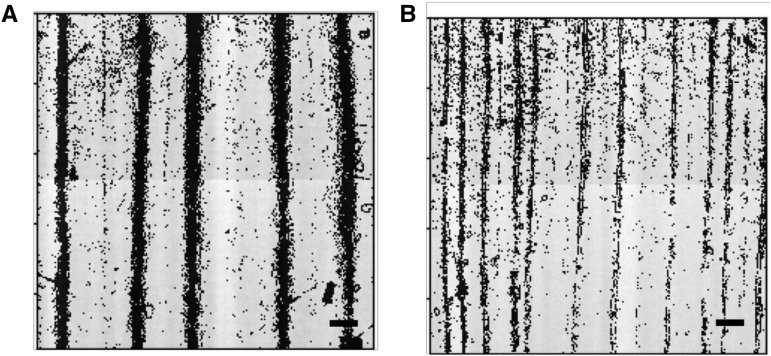
Grayscale images of leaf epidermal glue-impressions from two maize inbred lines showing extreme bulliform cell patterning phenotypes. (A) Inbred line MS153 shows 5 bulliform cell columns in this image, with an average width of 187.05 μm. (B) A374 has 11 bulliform cell columns with an average width of 63.57 μm. Scale bar 500 μm.

**Figure 3:**
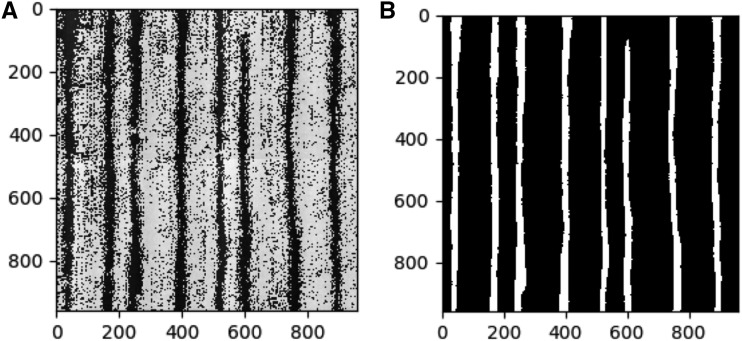
Segmentation output of U-nets from inbred line B79. (A) The raw image without annotation. (B) The segmentation map of the U-net output of the raw image in (A). In (B) white columns are bulliform cell columns; all other cells in the epidermal background are black. Each axis labels the pixels.

To survey the genetic diversity in maize bulliform cell patterning, leaf epidermal glue-impressions were obtained from the WiDiv panel, comprising 461 maize inbred lines grown in Maricopa, AZ, and San Diego, CA. Five glue-impressions per inbred line at each environment were sampled and three microscopic images were taken per glue-impression, for a total of 15,195 images. As shown in Figure 2, inbred lines comprising the WiDiv panel exhibit extreme variation in both bulliform column number and cell width (Table 1, Table S20). To enable faster computation, each image was then subdivided into four segments. The resulting 60,780 sub-images were input to CNNs (U-nets) for computational identification (segmentation) of bulliform cells from the leaf epidermal glue-impressions. An output segmentation map, *i.e.*, a binary grayscale image, was generated after the U-net segmented the raw images (Figure 3). The U-net model displayed an accuracy of 96.46% for bulliform column number, and 89.33% for bulliform column width.

**Table 1:** Phenotypic diversity and heritability of bulliform patterning traits assessed in this study

Trait	Number of lines	BLUPs in environments combined			BLUPs in Maricopa, AZ			BLUPs in San Diego, CA			Heritabilities		
		Mean	SD	Range	Mean	SD	Range	Mean	SD	Range	Environments combined	Maricopa, AZ	San Diego, CA
Column number	461	9.35	0.7	7.15-11.79	9.67	0.84	7.42-12.36	8.91	0.72	6.60-11.13	0.76 ± 0.024	0.86 ± 0.030	0.70 ± 0.066
Column width	461	103.51	10.21	80.33-138.28	103.52	12.18	70.27-146.61	101.2	13.67	70.64-148.77	0.71 ± 0.029	0.81 ± 0.044	0.81 ± 0.041

Both bulliform cell patterning traits were highly heritable, indicating that these bulliform cell patterning traits have a strong genetic underpinning and are amenable to GWAS. Specifically, heritabilities on a line-mean basis for column number and width were 0.76 and 0.71, respectively, across both environments, with plot-level heritability within each environment varying from 0.70 and 0.86 (Table 1).

### GWAS of bulliform cell patterning traits

**Figure 4:**
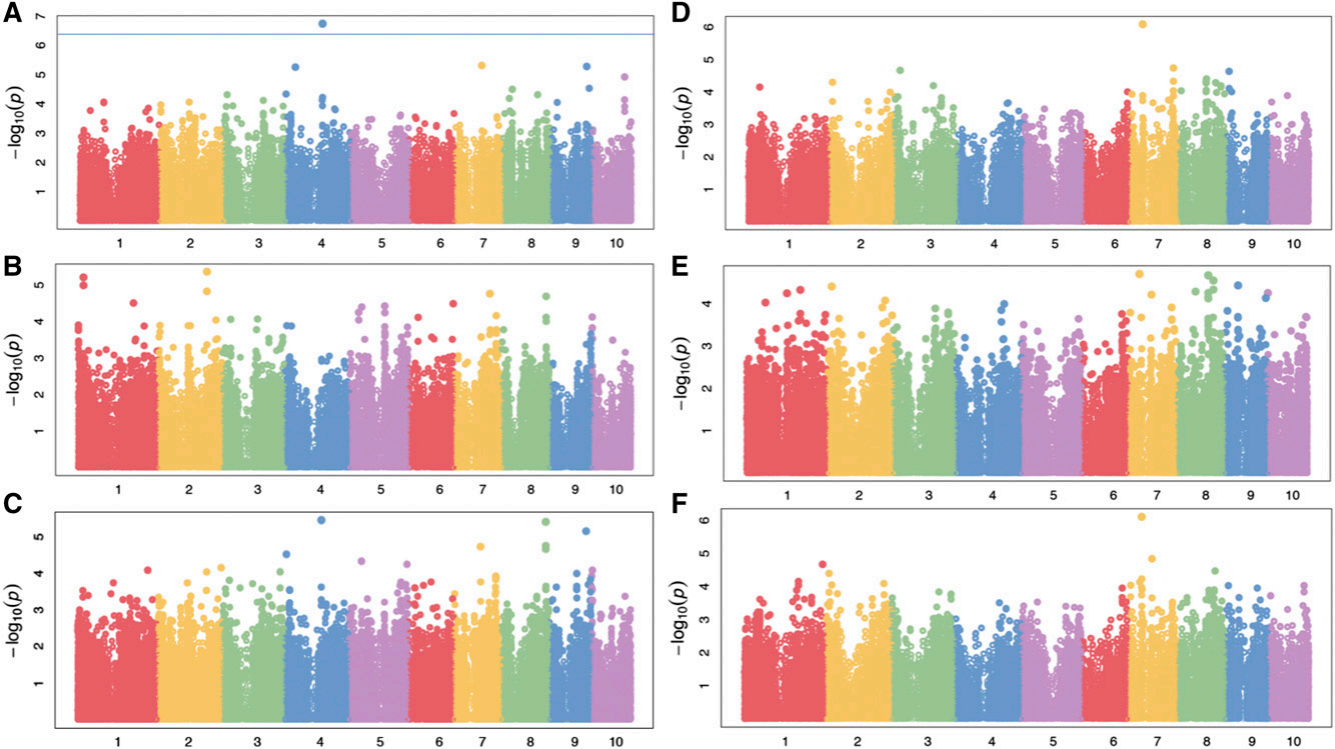
GWAS Manhattan plots for bulliform cell patterning traits. (A) Bulliform column number in Maricopa, AZ. The blue line indicates 0.05 FDR. (B) Bulliform column number in San Diego, CA. (C) Bulliform column number in both environments combined (Maricopa, AZ and San Diego, CA). (D) Bulliform column width in Maricopa, AZ. (E) Bulliform column width in San Diego, CA. (F) Bulliform column width in both environments combined (Maricopa, AZ and San Diego, CA).

The genetic architecture of bulliform cell patterning traits was investigated with the WiDiv panel. GWAS results individually from Maricopa, AZ, San Diego, CA, and combined results from both environments are summarized in Figure 4 (full datasets shown in Tables S21 – S26). A single SNP (located at 140,081,599 bp on chromosome 4, with raw p-values of $1.77\times 10^{-7},1.24\times 10^{-3},\;3.52\times 10^{-6}$ in AZ, CA, and both environments combined, respectively) is associated with bulliform column number at the 5% FDR level in the Maricopa environment. Although this same locus is also the top SNP (*i.e.*, most significant) associated with bulliform column number across environments, it is not significant at the 5% FDR level. In addition, this locus is not among the top SNPs for bulliform column number in San Diego, CA.

To search for candidate genes regulating bulliform column number, we investigated LD of the top SNP with nearby SNPs on chromosome 4; nine genes were identified within an 863.0 kb window having an$\;r^{2}$ greater than 0.5 with the top SNP (local LD decay shown in Figure S2). However, just one of these candidate loci (*Zm00001d051057*) is transcriptionally upregulated in the bulliform ontogenic zone (Table 2), the predicted site of bulliform cell patterning. *Zm00001d051057* is predicted to encode a HISTONE-LYSINE N-METHYLTRANSFERASE and is homologous to the Arabidopsis gene *ASH1-RELATED3 (ASHR3*). *ASHR3* encodes a SET-domain protein conferring histone H3 lysine-36 methylation, with implicated functions during regulation of stem cell division in the root apical meristem (Kumpf *et al.* 2014). We speculate that in maize, this *HISTONE-LYSINE N-METHYLTRANSFERASE* homolog may regulate cell division in bulliform column initial cells.

**Table 2:** Gene candidates identified in GWAS and differential expression analysis

Trait	Candidate Gene	Upregulated in bulliform ontogenic zone?	Maize Gene Name	Arabidopsis Gene Name
Column Number	Zm00001d051057	YES	*ASHR3*	*ASH1-RELATED 3*
Column Width	Zm00001d019696	YES	*CYCLIN10*	*CYCD3;2*
Column Width	Zm00001d019677	YES	*NA*	*VIER F-BOX PROTEIN 1*
Column Width	Zm00001d019688	YES	*NA*	*DEFECTIVE IN MERISTEM SILENCING 5*
Column Width	Zm00001d019681	YES	*CYCLIN-L1-1*	*ARGININE-RICH CYCLIN 1 CYTOCHROME OXIDASE*
Column Number	Zm00001d051055	NO	*CYTOCHROME C OXIDASE POLYPEPTIDE*	*CYTOCHROME OXIDASE*
Column Number	Zm00001d051062	NO	*GRPE PROTEIN HOMOLOG*	*CHLOROPLAST GRPE 1*
Column Number	Zm00001d051063	NO	*PHOSPHATIDYL-N-METHYLETHANOLAMINE N-METHYLTRANSFERASE*	*ARABIDOPSIS PHOSPHOLIPID N-METHYLTRANSFERASE*
Column Number	Zm00001d051061	NO	*ENHANCER OF RUDIMENTARY HOMOLOG*	*ARABIDOPSIS THALIANA ENHANCER OF RUDIMENTARY HOMOLOG*
Column Number	Zm00001d051065	NO	*NUCLEAR CAP-BINDING PROTEIN SUBUNIT 2*	*CAP-BINDING PROTEIN 20*

In the same 863.0 kb region near the bulliform column number SNP, there are five genes downregulated in the bulliform ontogenic zone (Table 2). These include *Zm00001d051065*, which encodes a homolog of the Arabidopsis *CAP-BINDING PROTEIN 20* that is implicated in epidermal patterning (Jäger *et al.* 2011), and a putative cell-cycle gene homolog (*Zm00001d051061*) (Gelsthorpe *et al.* 1997). These comprise additional candidate genes regulating bulliform cell patterning.

Our GWAS identified a single top locus (located at 50,129,023 bp on chromosome 7, with raw p-values of $8.11\times 10^{-7},\;7.77\times 10^{-7},2.09\times 10^{-4}$ in AZ, CA, and both environments combined, respectively) for bulliform cell column width (not significant at 5% FDR in any field environment). The most significant SNP in Maricopa, AZ, and in both environments combined, this SNP is also highly ranked in San Diego, CA. Among the 16 genes found to reside in a 1.93 Mb region spanned by SNPs having an$r^{2}$greater than 0.5 with this top SNP (local LD decay shown in Figure S3; high LD due to proximity to the centromere), four are transcriptionally upregulated in the bulliform ontogenic zone when compared to the bulliform maturation zone (Table 2). Notably, maize gene *Zm00001d019696* is predicted to encode a CYCLIN10 homolog, implicated to function during regulation of cell division. The Arabidopsis homolog CYCD3;2 mediates response to cytokinin, and regulates cell number in lateral organs (Dewitte *et al.* 2007). Other candidate genes for bulliform cell width include a second predicted cyclin (*CYCLIN-LI-1*), as well as *Zm00001d019677* and *Zm00001d019688*. *Zm00001d019677* is predicted to encode a maize homolog of the Arabidopsis F-box protein VIER F-BOX PROTEIN1, whereas *Zm00001d019688* is homologous to the Arabidopsis gene *DEFECTIVE IN MERISTEM SILENCING 5* (*DMS5*) that functions in RNA-directed DNA methylation (López Sánchez *et al.* 2016; Choudury *et al.* 2019). Intriguingly, the maize *ASHR3*-like gene, implicated above in our GWAS of bulliform row number, functions in histone methylation (Kumpf *et al.* 2014). These data suggest that bulliform cell patterning may be epigenetically regulated.

Despite the high heritability of the bulliform cell patterning traits described in this study, few statistically-associated GWAS hits are identified. Several factors may contribute to this phenomenon. For example, bulliform cell patterning may be conditioned by several to many loci with relatively small effects, which our mapping population may have insufficient statistical power to detect. In addition, these phenotypes could also be controlled by rare alleles (<1% minor allele frequency) in the population, which would likely not be in strong LD with the more common in frequency SNPs tested in GWAS. Lastly, extremely diverse environments may have dramatic effects on bulliform cell patterning phenotypes, thus why the strongest associations were mainly identified in the Maricopa environment. Plants grown in Maricopa, AZ, are predicted to undergo extreme water conservation responses, as compared to the same inbred lines cultivated in the milder climate of San Diego, CA. Specifically, the Pearson’s pairwise correlations between these two environments for column number and width are 0.60 and 0.56, respectively, which is suggestive of genotype-by-environment effects. Additional environmental replicates may help dissect the genotype-by-environment effects of this potentially genetically complex trait.

This study combines developmental analyses and stage-specific transcriptomics with the high-throughput microscopic phenotyping power enabled by machine learning, together with quantitative genetics and genomics, to investigate the genetic architecture of bulliform cell patterning. Although a microscopic phenotype, bulliform cell patterning is an important agronomic trait with implications in macroscopic phenotypes such as plant architecture and drought resistance. We identify five candidate genes in the regulation of bulliform column number and width. Future reverse genetic analyses, and transcriptomic studies of bulliform cell patterning mutants, can further investigate the roles of these candidate genes in this important yet understudied trait.
